# Familial Hypercholesterolemia: Real-World Data of 1236 Patients Attending a Czech Lipid Clinic. A Retrospective Analysis of Experience in More than 50 years. Part I: Genetics and Biochemical Parameters

**DOI:** 10.3389/fgene.2022.849008

**Published:** 2022-02-28

**Authors:** Veronika Todorovova, Tereza Altschmiedova, Michal Vrablik, Richard Ceska

**Affiliations:** Third Department of Medicine—Department of Endocrinology and Metabolism of the First Faculty of Medicine, Charles University and General University Hospital, Prague, Czechia

**Keywords:** familial hypercholesterolemia, familial defective apolipoprotein B-100, LDL-C, ApoB, ApoE isoform, Lp(a), statin, ASCVD

## Abstract

**Introduction:** The cause of familial hypercholesterolemia (FH) is defect in LDL receptor or familial defect of apolipoprotein B-100 (FDB) or, rarely, defect in proprotein convertase subtilisin/kexin type 9. Identification and treatment of patients with FH improves their prognosis. Our data represent retrospective analysis of 50 years of specialised care in our center.

**Patients and Methods:** A group of 1236 FH patients (841 women, 395 men; 993 study subjects and 243 relatives; mean age 44.8 ± 16.7 years) included 154 FDB patients followed at the Lipid Clinic of the General University Hospital in Prague since the mid-1960s to the present. Clinical diagnosis was based on the Dutch Lipid Clinic Network Criteria. Genetic analysis was performed using PCR-RFLP to detect FDB and apolipoprotein E (APOE) polymorphism. Biochemical data were collected and statistically analysed.

**Results:** At baseline, mean LDL-C and total cholesterol (TC) levels of all FH patients combined were 6.49 ± 1.92 mmol/L and 8.95 ± 1.95 mmol/L, respectively. Their LDL-C levels decreased to 3.26 ± 1.57 mmol/L and TC levels to 5.43 ± 1.69 mmol/L during follow-up. In the subgroup of LDL receptor-mediated FH (non-FDB) patients, baseline LDL-C and TC levels of 6.61 ± 1.95 mmol/L and 9.09 ± 1.97 mmol/L declined to 3.21 ± 1.60 mmol/L and 5.39 ± 1.72 mmol/L, respectively, during follow-up. In the FDB subgroup of patients, baseline levels of LDL-C and TC were 5.57 ± 1.46 mmol/L and 7.88 ± 1.58 mmol/L decreasing to 3.45 ± 0.24 mmol/L and 5.58 ± 1.37 mmol/L, respectively, during follow-up. Differences were also found in the effects of various APOE isoforms on lipid lowering. A significant decrease in lipid parameters was observed with the E2E2 isoform whereas a minimal decrease was seen with the E4E4 and E3E3 isoforms.

**Conclusion:** Whereas, overall, non-FDB patients had higher baseline lipid levels, these levels declined more appreciably compared with FDB patients during follow-up. Our retrospective analysis also found different effects of APOE isoforms on the decrease in lipid levels.

## Introduction

Familial hypercholesterolemia (FH) is an autosomal dominant inherited disorder characterised by elevated levels of low-density lipoprotein cholesterol (LDL-C) whose accumulation leads to the development of atherosclerotic cardiovascular disease (ASCVD); moreover, if not treated properly, it may result in premature death (Watts et al., 2016). It is estimated that, while there are 30 million FH patients worldwide, most of them are unaware of their condition (The FH Foundation, 2021). The prevalence of heterozygous FH (HeFH) is 1 per 200 to 250, with their LDL-C levels ranging between 4 and 13 mmol/L (Cuchel et al., 2014; Benn et al., 2016). In homozygous FH patients, the levels of LDL-C are >13 mmol/L and the prevalence of this rare disease is approximately 1 per 160,000 to 300,000 (Cuchel et al., 2014). The diagnosis of FH is established based on the Dutch Lipid Clinic Network Criteria (DLCNC) categorizing patients into definite (>8 points), probable (6–8 points), possible (3–5 points) and unlikely (<3 points) FH groups. The patients are assigned to their respective categories based on each individual´s family history, clinical history, physical examination, levels of LDL-C and, possibly, genetic testing (Benn et al., 2016). The patients are first asked to change their eating habits and increase physical activity. However, lifestyle changes are not always enough and treatment has to be enhanced pharmacologically. Familial hypercholesterolemia patients are most often treated with statins, a class of drugs highly effective in lowering LDL-C levels, especially when combined with ezetimibe (Gagné et al., 2002). A breakthrough in the treatment of FH came with the discovery of PCSK9 inhibitors shown to decrease LDL-C by ≥50% (Watts et al., 2020).

Variants of three genes are a major cause of FH. The most common of these mutations occur in the LDL receptor (*LDLR*) gene and can lead to ligand-binding dysfunction, impaired LDL transport or internalization, recycling, or complete receptor deficiency (Soutar and Naoumova, 2007; Cuchel et al., 2014). Likewise, FH can be caused by mutations in the apolipoprotein B (*APOB*) and the proprotein convertase subtilisin/kexin type 9 (*PCSK9*) (Vrablik et al., 2020). To the best of our knowledge, no mutation in the *PCSK9* gene in the Czech population has been reported to date. An important role is also played by the apolipoprotein E (*APOE*) gene, which affects the levels of LDL-C thus contributing to higher LDL-C levels in FH patients (Pirillo et al., 2017; Rashidi et al., 2017; Khalil et al., 2021).

A mutation in the *APOB* gene causes familial defective apolipoprotein B-100 (FDB), an autosomal dominant disease of lipid metabolism similar to LDL receptor-mediated FH (non-FDB) characterised by elevated plasma LDL-C levels (Vega and Grundy, 1986; Innerarity et al., 1987). The prevalence of FDB varies largely being, e.g., approximately 1 per 209 in Switzerland while the figure for Denmark is 1 per 883 (Miserez et al., 1994; Miserez and Muller, 2000; Benn et al., 2016). Familial defective apolipoprotein B-100 is caused by monogenic variants in the *APOB* gene where a single amino acid, arginine, at position 3527 is replaced, most frequently, by glutamine (p.R3527Q) and, rarely, by tryptophan (p.R3527W) or lysine (p.R3527L) or at position 3558 where arginine is replaced by cysteine (p. R3558C). This replacement leads to other protein conformations disrupting the binding of apolipoprotein B-100 (as a part of LDL particles) to LDLR (Brown and Goldstein, 1986; Whitfield et al., 2004).

The most common APOE isoform is E3E3 with the p.C112 and p.R158 variants (Ferrières et al., 1994; Eichner et al., 2002; Phillips, 2014). A less frequent isoform increasing LDL-C levels and contributing to the risk of developing Alzheimer’s disease is E4E4, i.e., the p.C112R and p.R158 variants (Huebbe and Rimbach, 2017; Muñoz et al., 2019). A rare isoform is E2E2 determined by the p.C112 and p.R158C variants.

Familial defective apolipoprotein B-100 is clinically almost indistinguishable from FH; it is easier to identify FDB genetically as a common monogenic variant R3527Q. Unlike FDB, FH can be caused by monogenic variants as well as polygenic forms encountered in approximately 20% of FH patients (Trinder et al., 2019). Although many studies have focused on numerous aspects of FH, data in the relevant literature about the individual FH subgroups and the differences between them are relatively scarce. Still, it is most likely that the clinical features, effect of treatment and inherent risks of the disease are significantly different between the non-FDB and FDB subgroups of patients.

The aim of this retrospective analysis was to analyse data of a large homogeneous group of patients diagnosed to have FH and followed in a single lipid center and, also, to show the benefits of therapy and the results obtained over the course of half of a century in specialised care. This large group was followed and processed in 2 different perspectives. This article (Part I) is focused on differences in the lipid profiles in subgroups of FH patients, i.e. FDB versus non-FDB patients, and in FH patients with different *APOE* genotypes. Concurrent article (Part II) by Altschmiedova et al. (2022) is focused on clinical symptomatology, i.e., on differences between the parameters in patients whose FH is already complicated by overt ASCVD and those without ASCVD in order to identify factors contributing to a complicated course of the disease.

## Patients

### Characteristics of Individuals Diagnosed With FH

A total of 1236 FH patients (841 women and 395 men; 993 study subjects, 243 relatives; mean age 44.8 ± 16.7 years) attending the Lipid Clinic of the General University Hospital in Prague, Czech Republic, were followed. The diagnosis of FH in our patients was based on the Dutch Lipid Clinic Network Criteria (DLCNC). Genetic analysis including FDB and APOE isoforms was performed in more than 76% of FH patients; however, a mutation in the *LDLR* gene was investigated in only ≥10% of these patients (Supplementary Figure S1). Risk factors and clinical complications are summarised in Table 1 and they are in more detail described in the article about clinical symptomatology by Altschmiedova et al. (2022) Enrolled in the retrospective analysis were patients, both pharmacotherapy-naïve and those treated pharmacologically, and their relatives.

**TABLE 1:** Clinical characteristics.

Risk factors, clinical complications	Percentage (%)
DM	6.47
Hypertension	26.70
Smokers	31.39
Arcus lipoides corneae	3.80
Xanthalesma	4.61
Tendon xanthomas	3.32
CAD (MI included)	9.63
Stroke	2.51
PAD	2.59
Death	2.83

DM, diabetes mellitus; CAD, coronary artery disease; MI, myocardial infarction; PAD, peripheral arterial disease.

The first patients of this retrospective analysis have been followed since the mid-1960s when diagnosed with FH based on their clinical symptoms; complete biochemical and genetic data have been available here since 1974. The follow-up has continued to date with the latest biochemical values recorded in late 2020.

## Materials and Methods

### Biochemical Analysis

Blood samples were collected from study subjects. The serum levels of total cholesterol (TC), HDL-cholesterol (HDL-C) and triglycerides (TG) were measured enzymatically on automated analysers (Modular P800, Roche, Basel, Switzerland and UniCel DxC 880i Beckman Coulter, Brea, CA, United States). LDL-C was calculated using the Friedewald formula whereas apolipoprotein B (APOB) and lipoprotein (a) [Lp(a)] were measured by nephelometry and immunonephelometry.

### Genetic Analysis

Genomic DNA was isolated from peripheral blood collected into EDTA-anticoagulated tubes using the salting out method proposed by Miller et al., 1998. The concentration and purity of DNA were determined using a spectrophotometer (A260/A280; BioPhotometer Eppendorf 6131, Eppendorf, Germany).

The p.R3527Q (*MluI*) and p.R3558C (*MspI*) variants in the *APOB* gene were detected by polymerase chain reaction (PCR). Oligonucleotides 5′-CTT ACT TGA ATT CCA AGA GCA CCC-3′ and 5′-TGT ACT CCC AGA GGG AAT ATA CGC-3′ were used as the primer set. *MluI* and *MspI* as restriction enzymes were used in PCR-restriction fragment length polymorphism (PCR-RFLP) analysis. Fragments were subsequently separated and visualised by electrophoresis on GelRed-stained 4% agarose gel (MetaPhor-agarose: agarose = 3:1).

The *E2*, *E3*, *E4* variants in the *APOE* gene were detected by PCR. Here, oligonucleotides 5′-TCC AAG GAG CTG CAG GCG GCG CA-3′ and 5′-ACA GAA TTC GCC CCG GCC TGG TAC ACT GCC A-3′ were used as the primer set. *CfoI* as a restriction enzyme was used in PCR-RFLP analysis. Fragments were subsequently separated and visualised by electrophoresis on GelRed-stained 10% polyacrylamide gel.

Variants in the *LDLR* gene were analysed by Sanger sequencing. The specific primers (Supplementary Material S1) were designed according to the sequence of 18 *LDLR* exons. The sequencing reaction was performed using the BigDye Terminator v3.1 Cycle Sequencing Kit.

### Statistical Analysis of Baseline and Follow-Up Data

Statistical analysis was performed using STATISTICA 13 software (TIBCO Software Inc., Palo Alto, CA, United States). Values in the text, tables and figures are means ± standard deviation (SD). The level of statistical significance was set at 5%. Comparison of baseline vs. follow-up was done using the paired *t*-test. The lipid parameters of the two subgroups were compared using the two-sample *t*-test or, when comparing three groups, using analysis of variance (ANOVA).

## Results

**FIGURE 1:**
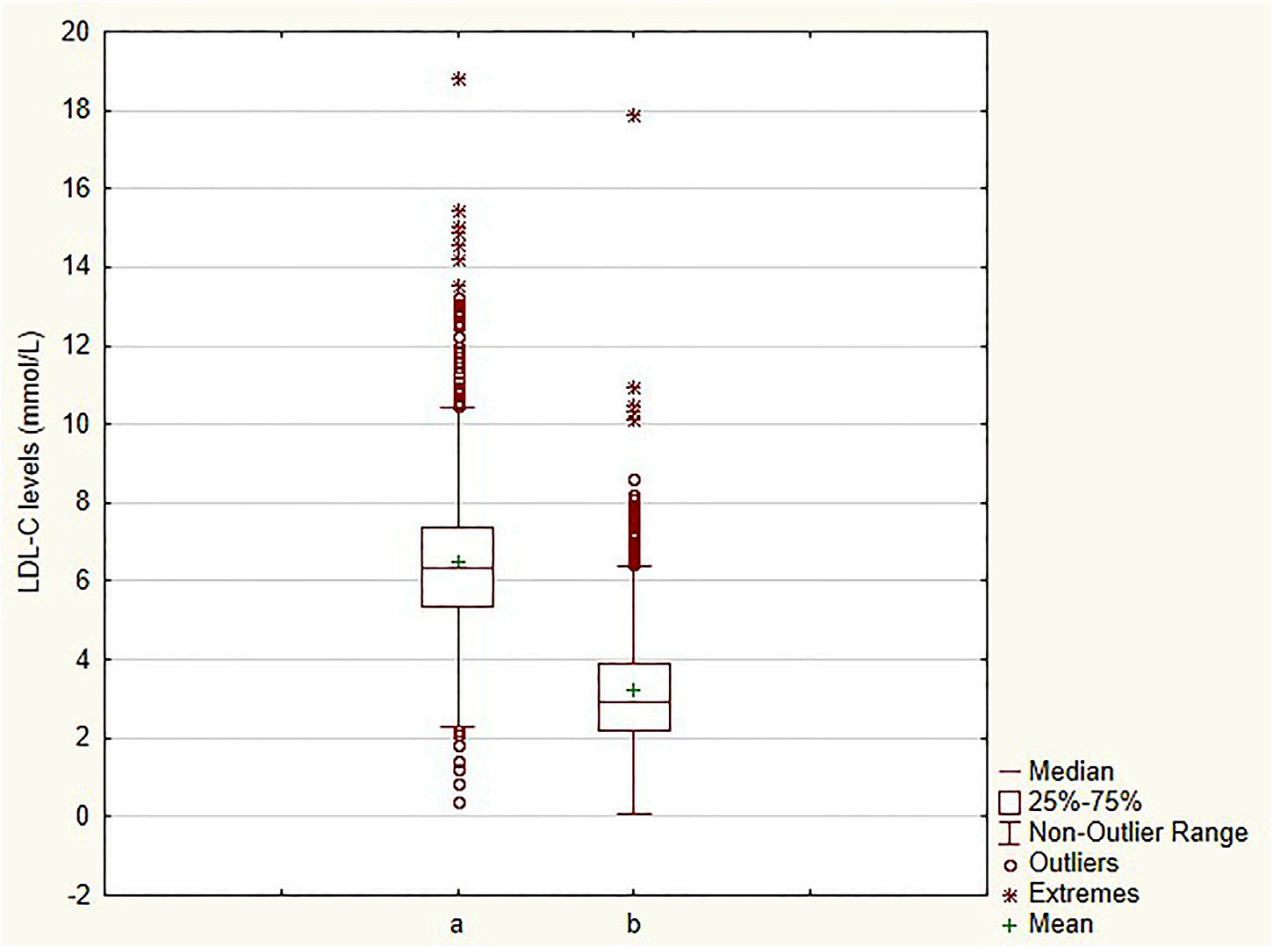
Comparison of LDL-C levels at baseline and at follow-up a—baseline; b—follow-up; LDL-C—low-density lipoprotein cholesterol.

An appreciable decrease in LDL-C levels, from 6.49 ± 1.92 mmol/L to 3.26 ± 1.57 mmol/L (∼49.8%) was observed (Figure 1) in all patients. Likewise, a 39.3% decrease, from 8.95 ± 1.95 mmol/L to 5.43 ± 1.69 mmol/L, in TC levels was seen (Supplementary Figure S2). The mean levels of other 2 parameters (TG and HDL-C) are clearly shown in Table 2. Major reductions were noted in APOB (−38.1%) and TG (−23.8%) levels (Supplementary Figure S3, S4). The decrease in HDL-C levels was only a small one (−6.6%) (Supplementary Figure S5). The differences between the baseline and follow-up levels of the parameters investigated were statistically significant (*p* < 0.001) except for Lp(a) whose levels remained almost unaltered throughout the retrospective analysis (Supplementary Figure S6).

**TABLE 2:** Baseline and follow-up lipid levels of FH cohort.

Parameter	Number of patients	Baseline	Follow-up	Difference (%)	*p*-value
		Mean ± SD	Mean ± SD		
LDL-C (mmol/L)	1,049	6.49 ± 1.92	3.26 ± 1.57	−49.8	*p* < 0.001
TC (mmol/L)	1,118	8.95 ± 1.95	5.43 ± 1.69	−39.3	*p* < 0.001
APOB (g/L)	184	1.76 ± 0.56	1.09 ± 0.56	−38.1	*p* < 0.001
TG (mmol/L)	1,108	1.81 ± 1.13	1.38 ± 0.78	−23.8	*p* < 0.001
HDL-C (mmol/L)	1,092	1.67 ± 0.46	1.56 ± 0.46	−6.6	*p* < 0.001
Lp(a) (g/L)	284	0.56 ± 0.74	0.59 ± 0.74	5.4	*p* = 0.2706

SD, standard deviation.

Based on the division of our patients into subgroups according to the use (Y) or non-use (N) of therapy, statistically significant differences (*p* < 0.005) were found between the subgroups in the baseline levels of LDL-C, TC, APOB, TG, and Lp(a). However, statistically significant differences (*p* < 0.001) between the subgroups were found during follow-up in their LDL-C, TC, APOB, and HDL-C levels. When comparing the on-therapy levels of these parameters with baseline, the biggest decreases—except for Lp(a)—were noted in the groups not initiating their therapy until the start of the analysis (N/Y). In this particular group, LDL-C, TC and APOB levels dropped by as much as 55.7, 44.8 and 45.4%, respectively (Table 3). A smaller decline (−26.4%) in the N/Y group occurred in TG levels. Decreases in the levels of lipid parameters were likewise observed in the group of patients on pre-existing therapy (Y/Y) whose LDL-C, TC and APOB levels decreased by 49.6, 38.2 and 37.0%, respectively. The Y/Y group showed a smaller decrease in TG levels (−23.4%). As to HDL-C levels, similar to all study parameters except for Lp(a), there was an obvious decrease in the group of patients not receiving therapy throughout the analysis (N/N). The decreases in the levels of LDL-C, TC, APOB, TG and HDL-C were significant in all groups of patients (categorised by their therapy) throughout the analysis (*p* < 0.05).

**Table 3:** Distribution of FH patients by treatment and effect of treatment on lipid levels.

Parameter	Group	Baseline			Follow-up					
		N	Mean ± SD	*p*	N	Mean ± SD	*p*	N	Diference (%)	*p*
LDL-C (mmol/L)	Y/Y	167	5.76 ± 1.93	*p* < 0.001	166	2.89 ± 1.13	*p* < 0.001	160	−49.6	*p* < 0.001
	N/Y	678	6.83 ± 1.80		678	3.01 ± 1.37		660	−55.7	
	N/N	172	6.35 ± 2.07		97	5.48 ± 2.02		93	−10.7	
TC (mmol/L)	Y/Y	175	8.15 ± 1.98	*p* < 0.001	169	5.03 ± 1.31	*p* < 0.001	169	−38.2	*p* < 0.001
	N/Y	699	9.32 ± 1.83		689	5.16 ± 1.49		689	−44.8	
	N/N	177	8.72 ± 2.07		102	7.84 ± 1.90		102	−8.8	
APOB (g/L)	Y/Y	97	1.57 ± 0.51	*p* < 0.001	65	0.98 ± 0.34	*p* < 0.001	43	−37.0	*p* < 0.001
	N/Y	316	1.86 ± 0.51		190	0.99 ± 0.39		86	−45.4	
	N/N	89	1.85 ± 0.65		40	1.69 ± 0.79		14	−7.9	
TG (mmol/L)	Y/Y	174	1.86 ± 1.17	*p* = 0.003	169	1.41 ± 0.69	*p* = 0.792	168	−23.4	*p* = 0.026
	N/Y	690	1.85 ± 1.17		688	1.37 ± 0.80		680	−26.4	
	N/N	177	1.54 ± 0.84		102	1.38 ± 0.84		102	−11.8	
HDL-C (mmol/L)	Y/Y	174	1.63 ± 0.39	*p* = 0.536	169	1.51 ± 0.40	*p* < 0.001	168	−6.9	*p* < 0.001
	N/Y	687	1.67 ± 0.45		682	1.53 ± 0.44		671	−8.2	
	N/N	175	1.67 ± 0.50		102	1.77 ± 0.54		101	1.9	
Lp(a) (g/L)	Y/Y	154	0.61 ± 0.66	*p* = 0.003	47	0.74 ± 0.67	*p* = 0.229	47	7.7	*p* = 0.921
	N/Y	553	0.44 ± 0.60		181	0.57 ± 0.80		180	4.8	
	N/N	113	0.39 ± 0.62		14	0.38 ± 0.39		13	12.1	

Y/Y, on treatment at baseline and throughout the analysis; N/Y, no treatment at baseline/on treatment throughout the analysis; N/N, no treatment at baseline and throughout the analysis; N, number of patients; SD, standard deviation; p, *p*-value.

Another division of our population of FH patients was based on genetic analysis of *APOE* polymorphisms by individual APOE isoforms, where differences in the levels of individual lipid parameters throughout the analysis were compared (Table 4). The biggest decreases in the levels of lipid parameters were seen in patients with the E2E2 isoform, being 75.3, 59.0, 53.5, 56.2, 17.6, and 36.7% in LDL-C, TC, APOB, TG, HDL-C and Lp(a) levels, respectively. In patients with the other isoforms, the decreases in LDL-C, TC, APOB and TG levels were within the ranges of 49.0–57.8%, 36.8–43.9%, 32.3–57.3%, and 22.4–39.4%, respectively, while HDL-C levels remained almost unchanged. A decline in Lp(a) levels was only seen in patients with the E2E2 isoform. The decrease was significant (*p* < 0.001) in only TG levels during follow-up.

**TABLE 4:** Patients with specific APOE isoforms and effect of treatment on lipid levels.

Parameter	Group	Baseline			Follow-up					
		N	Mean ± SD	*p*	N	Mean ± SD	*p*	N	Diference (%)	*p*
LDL-C (mmol/L)	E3E4	219	6.40 ± 1.80	*p* = 0.753	212	3.09 ± 1.36	*p* = 0.034	202	−50.9	*p* = 0.615
	E2E3	63	6.58 ± 2.22		59	3.27 ± 1.45		58	−50.3	
	E3E3	651	6.49 ± 1.92		603	3.32 ± 1.65		588	−49.0	
	E2E4	9	6.03 ± 2.36		11	2.45 ± 0.77		9	−57.8	
	E4E4	22	5.94 ± 1.97		21	3.02 ± 1.26		21	−49.0	
	E2E2	4	6.51 ± 1.30		6	1.77 ± 0.50		4	−75.3	
TC (mmol/L)	E3E4	230	8.90 ± 1.86	*p* = 0.644	217	5.29 ± 1.46	*p* = 0.126	217	−40.5	*p* = 0.202
	E2E3	65	8.93 ± 2.22		61	5.42 ± 1.62		61	−39.6	
	E3E3	668	8.94 ± 1.97		619	5.48 ± 1.78		619	−38.9	
	E2E4	11	8.29 ± 2.07		11	4.65 ± 0.84		11	−43.9	
	E4E4	22	8.53 ± 1.98		21	5.38 ± 1.47		21	−36.8	
	E2E2	6	9.82 ± 1.98		6	4.02 ± 0.60		6	−59.0	
APOB (g/L)	E3E4	116	1.79 ± 0.53	*p* = 0.129	84	1.09 ± 0.46	*p* = 0.361	47	−37.7	*p* = 0.933
	E2E3	35	1.67 ± 0.62		24	1.13 ± 0.40		14	−32.3	
	E3E3	348	1.80 ± 0.54		182	1.08 ± 0.54		97	−39.8	
	E2E4	6	1.76 ± 0.68		3	0.81 ± 0.10		2	−45.5	
	E4E4	11	1.60 ± 0.34		7	0.78 ± 0.28		3	−57.3	
	E2E2	5	1.22 ± 0.24		2	0.60 ± 0.11		1	−53.5	
TG (mmol/L)	E3E4	228	1.86 ± 1.28	*p* < 0.001	217	1.44 ± 0.77	*p* = 0.485	215	−24.1	*p* < 0.001
	E2E3	65	1.77 ± 1.10		61	1.34 ± 0.54		61	−24.4	
	E3E3	662	1.73 ± 1.08		619	1.34 ± 0.83		613	−23.6	
	E2E4	11	2.30 ± 1.68		11	1.39 ± 0.76		11	−39.4	
	E4E4	22	1.76 ± 0.84		21	1.38 ± 0.72		21	−22.4	
	E2E2	6	4.20 ± 2.50		6	1.84 ± 0.53		6	−56.2	
HDL-C (mmol/L)	E3E4	225	1.71 ± 0.47	*p* = 0.141	214	1.58 ± 0.45	*p* = 0.391	209	−8.3	*p* = 0.443
	E2E3	65	1.59 ± 0.51		60	1.53 ± 0.50		60	−5.1	
	E3E3	662	1.67 ± 0.45		616	1.56 ± 0.47		610	−7.1	
	E2E4	11	1.52 ± 0.53		11	1.57 ± 0.45		11	3.3	
	E4E4	22	1.85 ± 0.49		21	1.78 ± 0.55		21	−4.5	
	E2E2	6	1.73 ± 0.41		6	1.43 ± 0.26		6	−17.6	
Lp(a) (g/L)	E3E4	199	0.52 ± 0.66	*p* = 0.219	61	0.66 ± 0.73	*p* = 0.361	60	−3.8	*p* = 0.692
	E2E3	51	0.32 ± 0.45		12	0.20 ± 0.18		12	10.0	
	E3E3	569	0.44 ± 0.56		173	0.53 ± 0.63		173	13.2	
	E2E4	9	0.42 ± 0.46		4	0.59 ± 0.95		4	51.3	
	E4E4	19	0.48 ± 0.62		5	0.94 ± 0.89		5	3.5	
	E2E2	6	0.16 ± 0.10		1	0.19 ± 0.00		1	−36.7	

N, number of patients; SD, standard deviation; *p*, *p*-value.

A total of 1008 FH patients were genetically tested. Familial defective apolipoprotein B-100 was detected in 154 patients (mean age 40.8 ± 18.1 years; 107 women and 47 men; 117 study subjects and 37 relatives). One of these patients was diagnosed as FDB homozygote. The rest of genetically tested FH patients group consisted of 854 patients (557 women and 277 men; 686 study subjects and 168 relatives) supposed to have mutation in *LDLR* gene (non-FDB) (mean age 44.8 ± 16.0 years). Five patients were diagnosed with homozygous FH due to a mutation in the *LDLR* gene. Overall, another 228 study subjects met the DLCN criteria for FH but genetic testing was not performed and, thus, these subjects were excluded from further analysis.

One of the main subgroups within our study participants was that of non-FDB patients where significant decreases in the levels of LDL-C were noted in 754 patients (−51.1%); TC, in 795 patients (−40.7%); APOB, in 132 patients (−40.7%) and TG, in 788 patients (−24.4%). In 781 non-FDB patients, HDL-C levels declined by a mere 7.6% whereas Lp(a) levels remained almost unaltered. The second subgroup consisted of 154 patients with FDB. Their mean values of lipid parameters, both baseline and follow-up, are given in more detail in Table 5. Among the FDB patients, appreciable decreases in LDL-C were seen in 120 patients (−37.7%), TC in 131 patients (−30.3%), APOB in 26 patients (−29.2%), and TG in 130 patients (−24.0%). In 127 patients, HDL-C levels decreased by 5.9% during follow-up.

**TABLE 5:** FDB and non-FDB patients and effect of treatment on lipid levels.

Parameter	FDB	Baseline			Follow-up					
		N	Mean ± SD	*p*	N	Mean ± SD	*p*	N	Diference (%)	*p*
LDL-C (mmol/L)	+	148	5.57 ± 1.46	*p* < 0.001	124	3.45 ± 0.24	*p* = 0.117	120	−37.7	*p* < 0.001
	−	812	6.61 ± 1.95		779	3.21 ± 1.60		754	−51.1	
TC (mmol/L)	+	153	7.88 ± 1.58	*p* < 0.001	131	5.58 ± 1.37	*p* = 0.252	131	−30.3	*p* < 0.001
	−	840	9.09 ± 1.97		795	5.39 ± 1.72		795	−40.7	
APOB (g/L)	+	85	1.53 ± 0.37	*p* < 0.001	43	1.13 ± 0.38	*p* = 0.448	26	−29.2	*p* = 0.023
	−	430	1.83 ± 0.56		255	1.07 ± 0.51		132	−40.7	
TG (mmol/L)	+	152	1.40 ± 0.98	*p* < 0.001	131	1.07 ± 0.51	*p* < 0.001	130	−24.0	*p* = 0.251
	−	833	1.86 ± 1.17		795	1.42 ± 0.83		788	−24.4	
HDL-C (mmol/L)	+	151	1.68 ± 0.47	*p* = 0.960	129	1.60 ± 0.46	*p* = 0.316	127	−5.9	*p* = 0.455
	−	831	1.68 ± 0.46		790	1.55 ± 0.47		781	−7.6	
Lp(a) (g/L)	+	133	0.40 ± 0.45	*p* = 0.229	33	0.65 ± 0.62	*p* = 0.350	33	61.2	*p* < 0.001
	−	715	0.46 ± 0.60		217	0.54 ± 0.66		216	1.0	

+, FDB; −, non-FDB; N – number of patients; SD, standard deviation; p, *p*-value.

A comparison of non-FDB and FDB patients revealed significant differences (*p* < 0.001) in their baseline levels of LDL-C, TC, APOB and TG whereas the difference versus follow-up levels was significant (*p* < 0.001) only in TG levels. Statistically significant differences (*p* < 0.05) were found between the baseline and follow-up levels of LDL-C, TC, APOB and Lp(a) in both, non-FDB and FDB, subgroups of patients.

At the end of the day, we would like to present the least favourable mean levels of Mr. and Mrs. FH and FDB in our analysis.

• Those of Mr. and Mrs. FH in our group are as follows: LDL-C 6.61 mmol/L, TC 9.09 mmol/L, APOB 1.83 g/L, TG 1.86 mmol/L, HDL-C 1.68 mmol/L and Lp(a) 0.46 g/L and
• Those of Mr. and Mrs. FDB in our group are as follows: LDL-C 5.57 mmol/L, TC 7.88 mmol/L, APOB 1.53 g/L, TG 1.40 mmol/L, HDL-C 1.68 mmol/L and Lp(a) 0.40 g/L.

## Discussion

What makes our retrospective analysis actually important is that our data were collected from a large group of more than 1,000 patients with FH attending a single lipid clinic. A positive finding of the long-term follow-up of patients in our center were decreases in the levels of LDL-C by more than 50%, which were not only statistically significant, but, also, clinically beneficial. Of no less importance was the decrease (by as much as 38%) in LDL-C levels in our FDB patients. As suggested by earlier reports, the levels of lipid parameters in FBD patients are generally lower than in those with LDL receptor-mediated FH (Gaffney et al., 2002; Vohnout et al., 2003; Fouchier et al., 2004). Similarly, the disorder diagnosed in our FDB homozygous patient was not as severe as that seen in homozygous individuals with receptor-mediated disorder. This may be explained by the APOE-regulated clearance of very low-density lipoprotein (VLDL) and intermediate-density lipoprotein (IDL) particles in FDB patients and the interaction between APOB and LDLR, important for the conversion of IDL to LDL-C (Gaffney et al., 2002; Vohnout et al., 2003).

Our analysis is a retrospective one whose first participants were receiving specialised care in a Prague-based clinic headed by Josef Šobra, with their data collection starting as early as 1960s (Šobra, 1970). Long-term care of these patients succeeded in reducing their cardiovascular risk and the clinic continues to provide individual care to each FH patient. While some of the patients have been taken care of for over 30 years, others have been attending the facility for less than 2 years; nonetheless, their personalised treatment plans have been shown to be beneficial in the long term. This explains the absence of statistically analysed data from the above period. This is partly due to the different numbers of patients and amount of analysed data in the individual subgroups of patients, with some of them referred to other physicians using different procedures, approaches and requirements for lipid parameter determination. However, the differences in the amount of data analysed and presented here are mainly due to FH patients referred from general practitioners to specialised centers; the result is some patients had incomplete baseline data while baseline blood sampling had not been performed in others. Another reason for the incompleteness data of some patients is only one value of some of the lipid parameters was obtained before the patient decided to discontinue follow-up.

A limitation of our analysis is the composition of our entire FH group consisting predominantly of patients attending a lipid clinic with only a small proportion being their family members. While not usual in other countries (Bhatnagar et al., 2000; Jarauta et al., 2016), a small number of relatives is a typical feature in the Czech Republic.

As expected, the differences in the decreases in lipid parameters between the untreated versus treated groups seen during the analysis in our lipid clinic were statistically significant. Nonetheless, the group of patients receiving treatment from practitioners prior to initiation of therapy by a lipid specialist also showed an appreciable decrease in their lipid levels, similar to that seen in patients not starting therapy before admission to our center. The implication is that targeted and proper management of FH patients is of crucial importance and, despite the undeniable role of general practitioners, tailored and specific care provided in lipid clinics is more effective and beneficial.

While it is difficult to identify a specific therapeutic strategy for over more than 50 years, generally, the treatment copied the availability and development of pharmacotherapy. It can be clearly stated that, until 1990, the mainstay of therapy of FH were cholestyramine and colestipol. Since 1990, treatment of FH has been based on statins (always the most efficient statin available, i.e., lovastatin, simvastatin, atorvastatin and rosuvastatin). A combination with ezetimibe has been used since the beginning of the 21st century and the monoclonal antibodies evolocumab and alirocumab have been available since 2018.

Patients with the rare *APOE E2E2* genotype showed an obviously major drop in the levels of LDL-C, TC and TG corresponding the metabolic processing of the E2E2 isoform, where particle clearance does not occur through binding to LDLR but through the LDL-related receptor and heparin sulfate proteoglycans (Phillips, 2014). A similar major decrease was observed in patients with the E2E4 isoform, processed partly in the same way as the above E2E2 isoform. In FH patients, the decreases seen with the E3 and E4 isoforms were smaller, a fact possibly attributable to the clearance of APOE *via* LDLR where binding may be impaired due to the high frequency of *LDLR* gene mutations in FH patients.

Another parameter assessed in our study were Lp(a) levels not showing significant changes in some of our study subgroups. This may be partly explained by the fact that analysis of Lp(a) levels was undertaken in a period when no therapy to modify Lp(a) levels was available yet.

The relationship between high Lp(a) levels and proprotein convertase subtilisin/kexin type 9 (PCSK9) was not investigated until 2018, when Sun et al. reported their data obtained from patients with heterozygous FH; it has been shown only recently that Lp(a) levels can be decreased with the use of PCSK9 inhibitors (Sun et al., 2018). PCSK9 inhibitors were approved for clinical use in the Czech Republic in 2018; hence, the introduction of PCSK9 inhibitors is not significantly reflected in our analysis.

### Limitation of Retrospective Analysis

Despite their long-term follow-up, a small group of patients has not had genetic testing, with their diagnosis established solely using the DLCNC. As a result, some of these patients could not be conclusively identified as actually being or not being FDB patients. The relatively small number of mutations detected in the *LDLR* gene is due to the fact that the sequencing technique developed by Sanger was adopted by our lipid clinic only recently. Besides, the technique is also more time-consuming than those of PCR restriction fragment length polymorphism (RFLP) or real-time PCR detecting point mutations. The analysed *LDLR* gene region contains 18 exons which have to be sequenced separately when using Sanger’s technique. The proportion of *LDLR* gene mutation analyses is likely to increase in our clinic with the introduction of new generation sequencing (NGS) techniques in the years to come.

Our retrospective analysis provides initial data obtained from a large group of patients attending a single lipid clinic and analysed in terms of the biochemical and genetic characteristics.

## Conclusion

Using a large group of patients with familial hypercholesterolemia, the present analysis reports data related to lipid and lipoprotein metabolism. The project was designed to assess changes in the levels of these parameters between baseline and follow-up in patients receiving personalised care admitted to our clinic. Our experience gained within the international ScreenPro FH project shows that patient surveillance and long-term follow-up are most beneficial as documented by Ceska et al., 2019. As an extension to the outcome of the present retrospective analysis, clinical data of our FH cohort are reported in Part II by Altschmiedova et al. (2022)

## Data Availability

The raw data supporting the conclusion of this article will be made available by the authors, without undue reservation.
