# Supplementary material for: Familial Hypercholesterolemia: Real-World Data of 1236 Patients Attending a Czech Lipid Clinic. A Retrospective Analysis of Experience in More than 50 years. Part I: Genetics and Biochemical Parameters
Source: Front Genet. 2022 Feb 28;13:849008. doi: 10.3389/fgene.2022.849008 (PMC8918685; doi:10.3389/fgene.2022.849008)
Supplement: Supplementary file 1 [file Table1.DOCX]

Supplementary Table 1 Primer sequences of 18 *LDLR* exons

| Primer | Primer sequence | Product lenght (bp) | Annealing Temperature (°C) |
| --- | --- | --- | --- |
| M13-LDLR-1F | 5´GTAAAACGACGGCCAGTGGAGTGGGAATCAGAGCTTCACGG | 435 | 60 |
| LDLR-1R | 5´CCATTACCCCACAAGTCTCCCAG |  |  |
| M13-LDLR-2F | 5´GTAAAACGACGGCCAGTTGGCAGGAAATAGACACAGGA | 294 | 56 |
| LDLR-2R | 5´ACCAGAAATTCAAGACCAGCCT |  |  |
| M13-LDLR-3F | 5´GTAAAACGACGGCCAGATATTGGCCAGGCTGGTCTTG | 802 | 62 |
| LDLR-3R | 5´GAGCAGGACCCCGTAGAGACA |  |  |
| M13-LDLR-4F | 5´GTAAAACGACGGCCAGGGTTCAGAGTCCATGGCCC | 635 | 62 |
| LDLR-4R | 5´GTTGTTGGAAATCCACTTCGGC |  |  |
| M13-LDLR-5F | 5´GTAAAACGACGGCCAGGGCCCTGCTTCTTTTTCTCTG | 268 | 57 |
| LDLR-5R | 5´CCCTCTGGCTTCACAAATCATT |  |  |
| M13-LDLR-6F | 5´GTAAAACGACGGCCAGCATTTGCATGCGTTCTTATGTG | 310 | 56 |
| LDLR-6R | 5´CCAAAACCCTACAGCACTCATG |  |  |
| M13-LDLR-7F | 5´GTAAAACGACGGCCAGGAGGTTGTAATGAGCCAAGGTTG | 302 | 58 |
| LDLR-7R | 5´AGCACACTTAACAGATGGGGAAAC |  |  |
| M13-LDLR-8F | 5´GTAAAACGACGGCCAGTTCGAAGGTGTGGGTTTTG | 322 | 58 |
| LDLR-8R | 5´TCAGGGGATATGAGTCTGTGC |  |  |
| M13-LDLR-9F | 5´GTAAAACGACGGCCAGTTTTCTGGGTGCCTCCTCTGG | 380 | 61 |
| LDLR-9R | 5´CTGAGGCAGGAGGAGAGAAGGG |  |  |
| M13-LDLR-10F | 5´GTAAAACGACGGCCAGATGATCTGCAGGTGAGCGTCG | 408 | 61 |
| LDLR-10R | 5´ATGCCCAGCCCACTAACCAGT |  |  |
| M13-LDLR-11F | 5´GTAAAACGACGGCCAGTGTTTCTTCCAGAATTCGTTG | 310 | 57 |
| LDLR-11R | 5´AACCTTCAGGGAGCAGCTTG |  |  |
| M13-LDLR-12F | 5´GTAAAACGACGGCCAGGGTGCTTTTCTGCTAGGTCCC | 357 | 58 |
| LDLR-12R | 5´TCACAACCAGTTTTCTGCGTTC |  |  |
| M13-LDLR-13F | 5´GTAAAACGACGGCCAGTAGTTGTGGAGAGAGGGTGG | 294 | 56 |
| LDLR-13R | 5´GAGGGTGGCCTGTGTCTCATC |  |  |
| M13-LDLR-14F | 5´GTAAAACGACGGCCAGCCCCAACCTTGAAACCTCCTT | 352 | 59 |
| LDLR-14R | 5´GGTACCCATTTGACAGATGAGCA |  |  |
| M13-LDLR-15F | 5´GTAAAACGACGGCCAGGGCCTCCCAAGGTCATTTGA | 351 | 58 |
| LDLR-15R | 5´CTCCGTGACCAAAATGTTCGTG |  |  |
| M13-LDLR-16F | 5´GTAAAACGACGGCCAGCATTTCTTGGTGGCCTTCCT | 224 | 57 |
| LDLR-16R | 5´AAAAAGTGAACAGGCCCAAC |  |  |
| M13-LDLR-17F | 5´GTAAAACGACGGCCAGCAAGGTTATGGTACGATGCCC | 333 | 58 |
| LDLR-17R | 5´TGGTCCCTTGAGGATCATATGC |  |  |
| M13-LDLR-18F | 5´GTAAAACGACGGCCAGGTACTCACCGTCTCCCTCTGGC | 298 | 59 |
| LDLR-18R | 5´ACAAAGCTCTGGCAGGCAATG |  |  |
