# Supplementary material for: Familial Hypercholesterolemia: Real-World Data of 1236 Patients Attending a Czech Lipid Clinic. A Retrospective Analysis of Experience in More than 50 years. Part I: Genetics and Biochemical Parameters
Source: Front Genet. 2022 Feb 28;13:849008. doi: 10.3389/fgene.2022.849008 (PMC8918685; doi:10.3389/fgene.2022.849008)
Supplement: Supplementary file 2 [file DataSheet1.PDF]

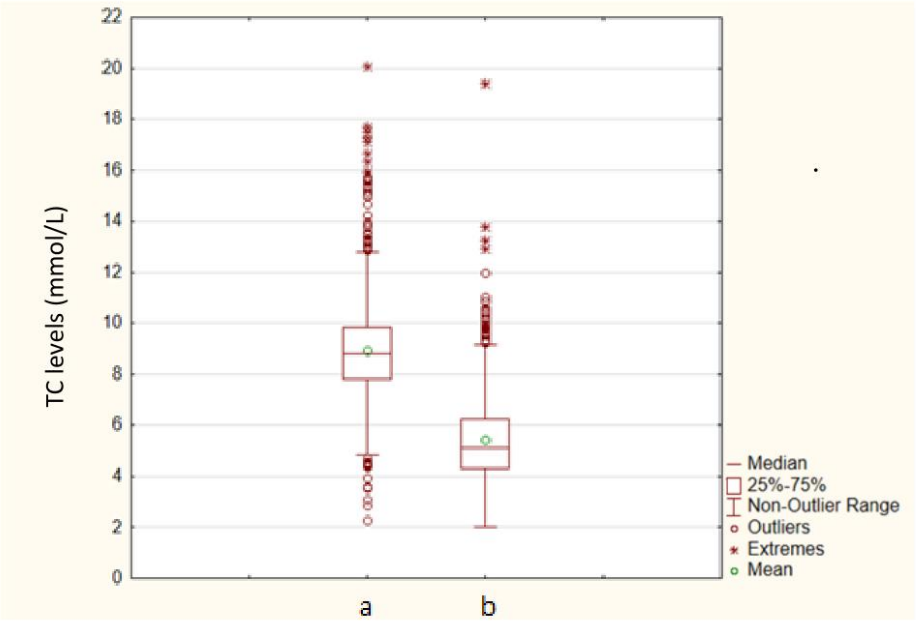

Figure 2 Comparison of TC levels at baseline and at end-of-study
a – baseline; b – end-of-study; TC – total cholesterol

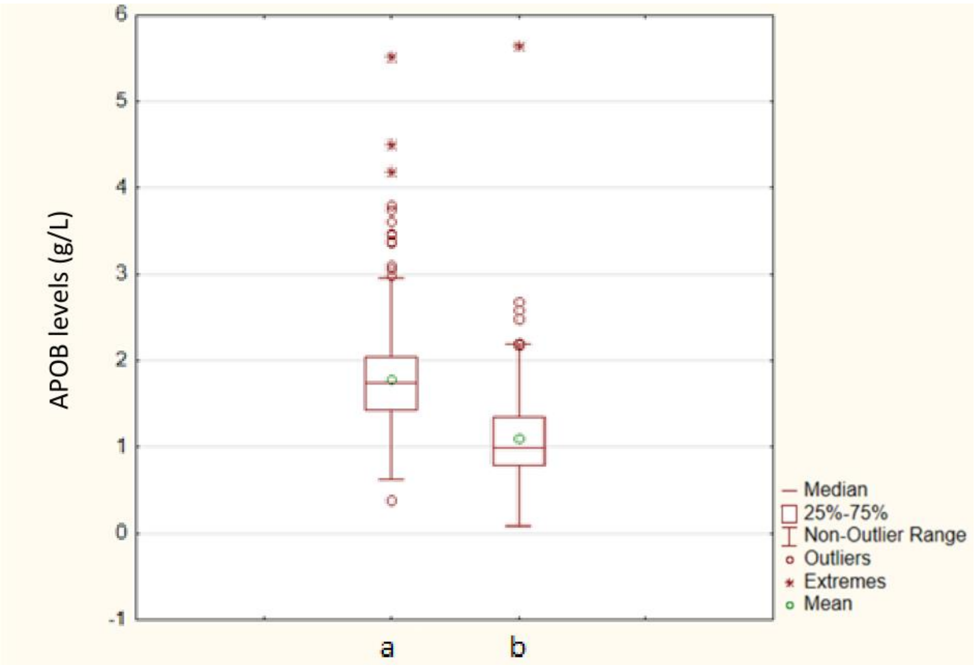

Figure 3 Comparison of APOB levels at baseline and at end-of-study
a – baseline; b – end-of-study; APOB – apolipoprotein B

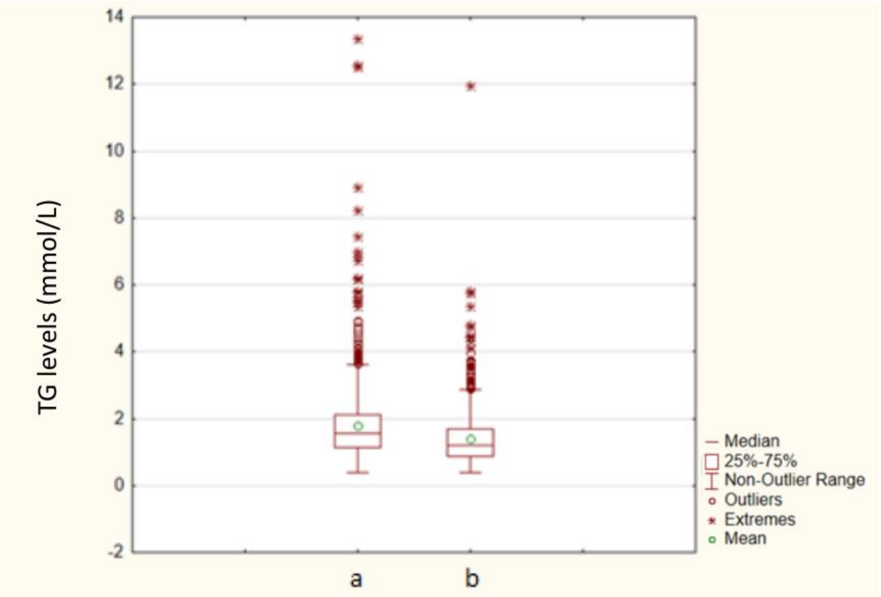

Figure 4 Comparison of TG levels at baseline and at end-of-study
a – baseline; b – end-of-study; TG – triglycerides

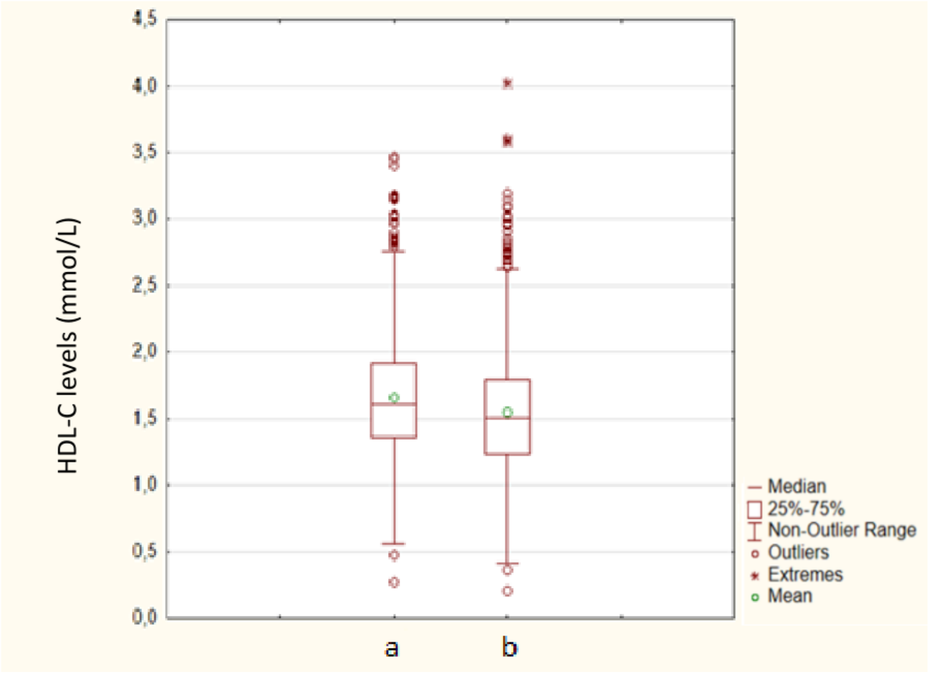

Figure 5 Comparison of HDL-C levels at baseline and end-of-study
a – baseline; b – end-of-study; HDL-C – high-density lipoprotein cholesterol

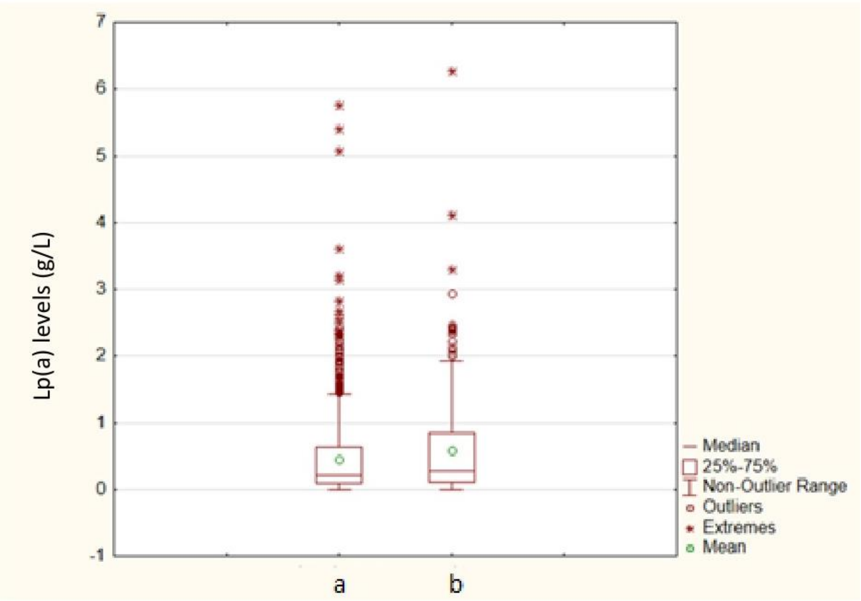

Figure 6 Comparison of Lp(a) levels at baseline and at end-of-study
a – baseline; b – end-of-study; Lp(a) – lipoprotein (a)
