# Supplementary figures and images for: Familial Hypercholesterolemia: Real-World Data of 1236 Patients Attending a Czech Lipid Clinic. A Retrospective Analysis of Experience in More than 50 years. Part I: Genetics and Biochemical Parameters
Source: Front Genet. 2022 Feb 28;13:849008. doi: 10.3389/fgene.2022.849008 (PMC8918685; doi:10.3389/fgene.2022.849008)

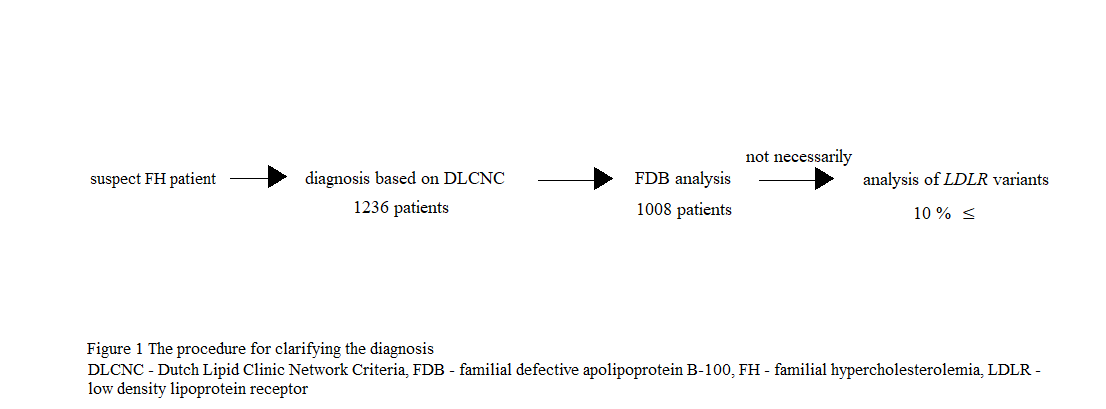

Supplement: Supplementary file 3 [file Image1.png]
